# Supplementary material for: Telomere-to-Telomere Haplotype-Resolved Genomes of Agrocybe chaxingu Reveals Unique Genetic Features and Developmental Insights
Source: J Fungi (Basel). 2024 Aug 25;10(9):602. doi: 10.3390/jof10090602 (PMC11433599; doi:10.3390/jof10090602)
Supplement: Supplementary file 1 [file jof-10-00602-s001.zip › jof-3162796-supplementary.pdf]

Supplementary material

Manuscript title: Telomere-to-telomere Haplotype-resolved Genomes of *Agrocybe chaxingu* Reveals Unique Genetic Features and Developmental Insights

Information

1. Supplementary Figures S1 to S5.

2. Supplementary Tables S1 to S12.

Figure S1.

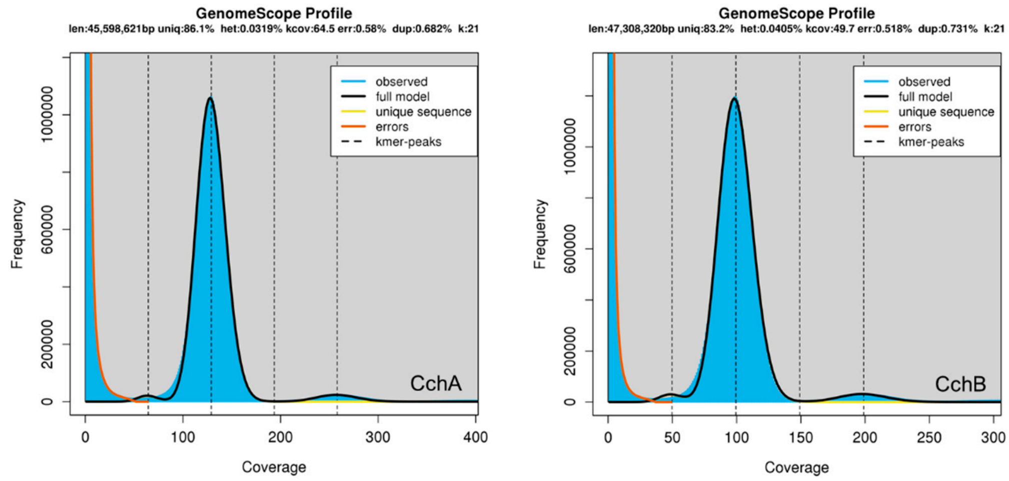

A **B**

Figure S1. The GenomeScope profiles of CchA (**A**) and CchB (**B**) based on 21 K-mer.

Figure S2.

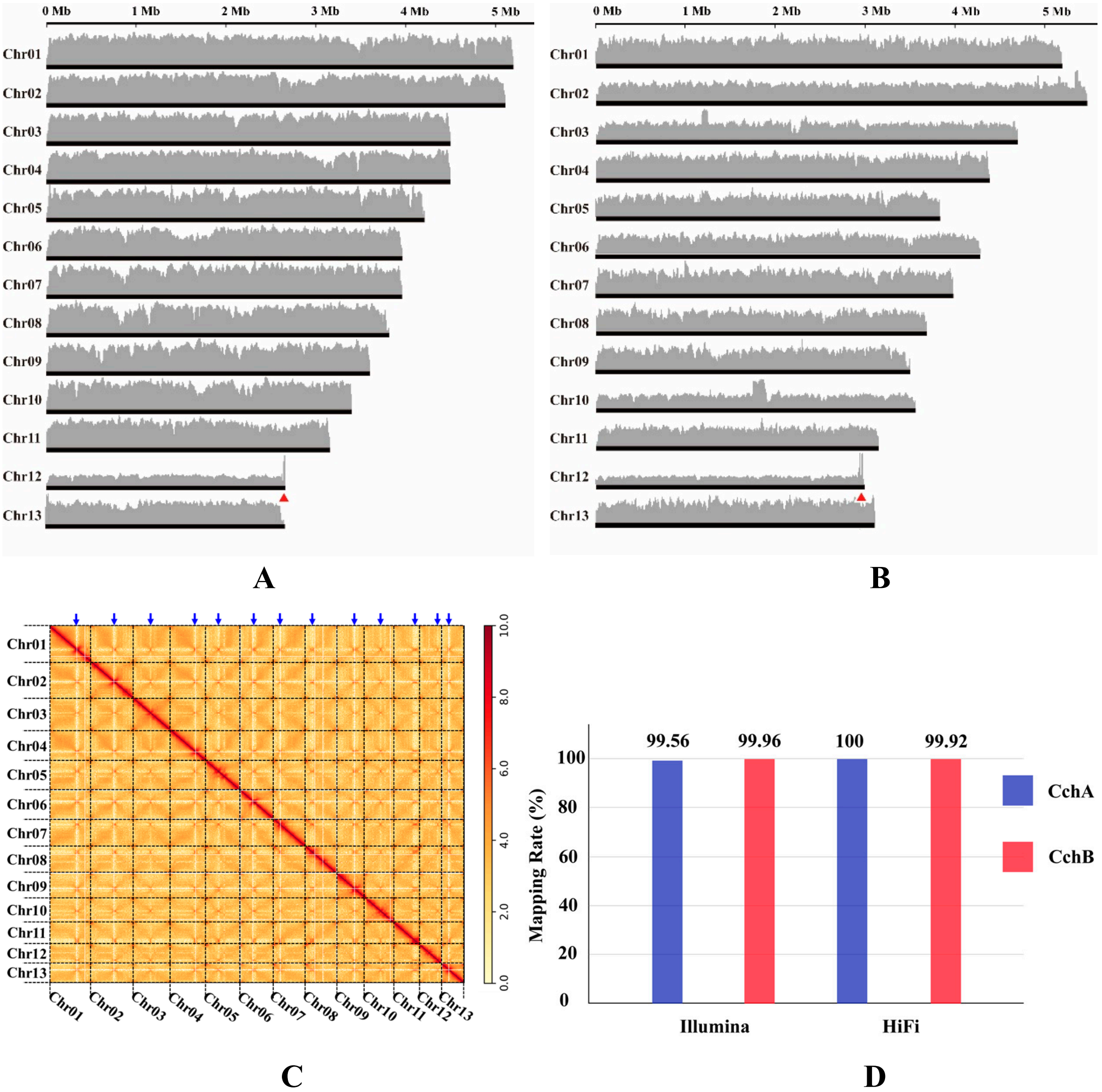

Figure S2. Overview of the *Agrocybe chaxingu* AS-5 assembly genome. (**A**) HiFi coverage of the 13 chromosomes of CchA. (**B**) HiFi coverage of the 13 chromosomes of CchB. (**C**) Hi-C interaction heatmap for the CchA genome. (**D**) Mapping rates of Illumina and HiFi reads in CchA and CchB. The red triangle represented rDNA. The blue arrows represented the potential centromeric regions.

Figure S3.

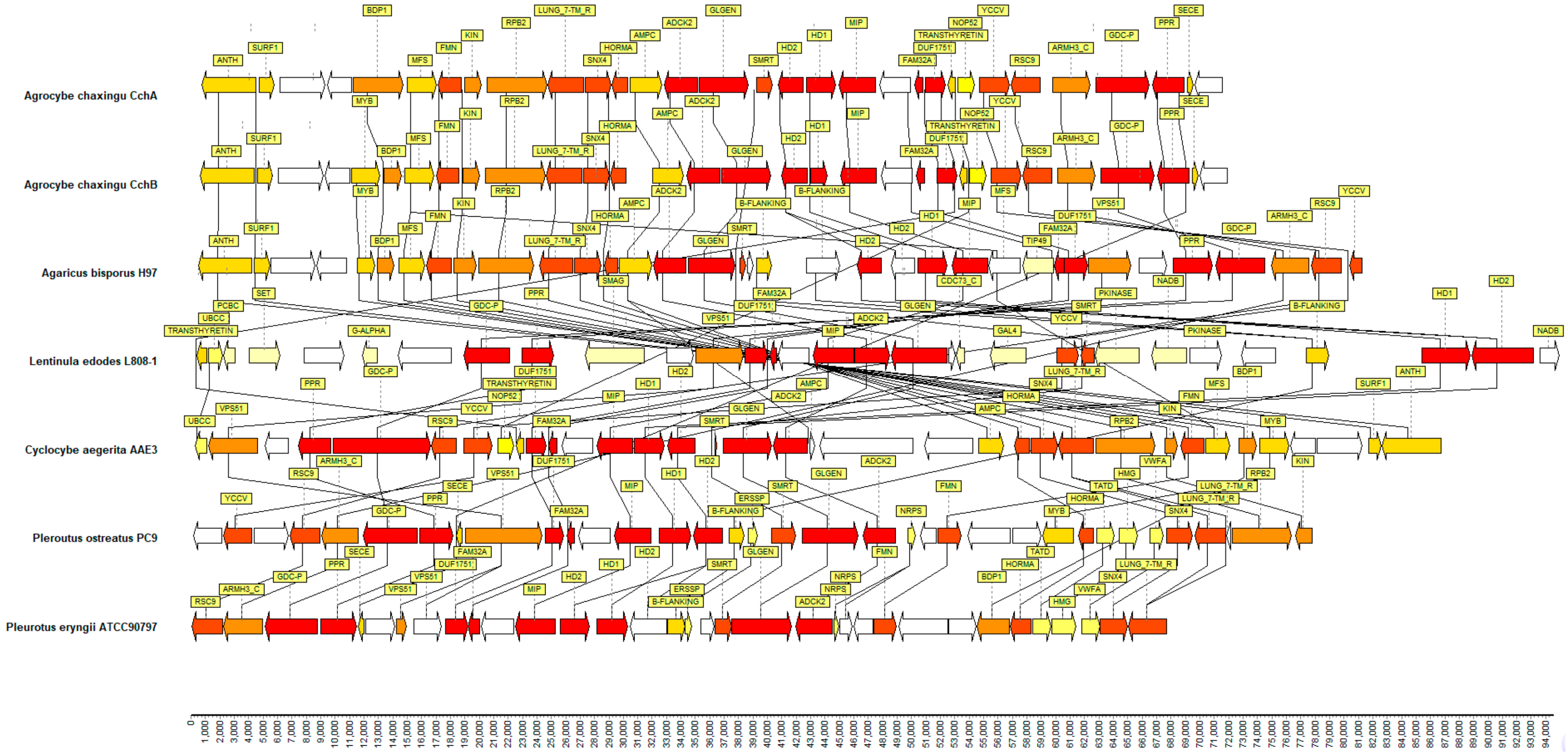

Figure S3. Alignment analysis of the mating type loci A in *Agrocybe chaxingu* strains CchA and CchB with those of other edible mushrooms. MIP: mitochondrial intermediate peptidase. HD1: Homeodomain1. HD2: Homeodomain2. B-FLANKING: β-flanking.

Figure S4.

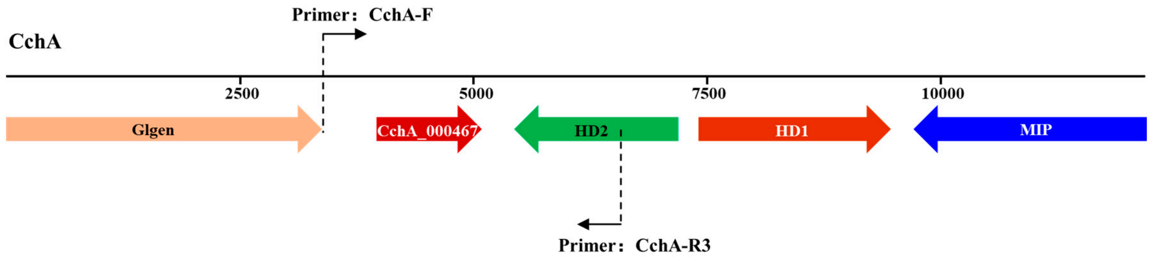

A

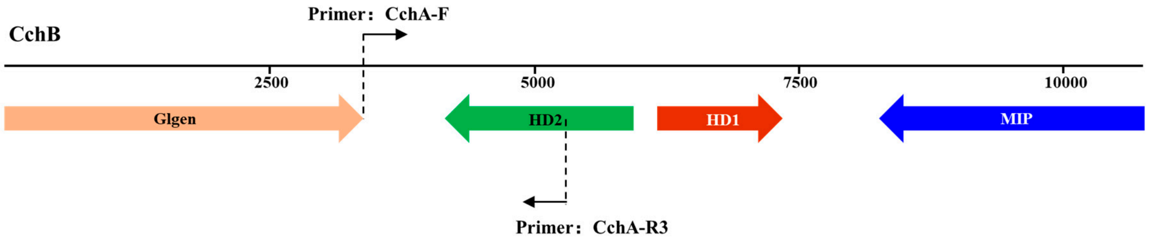

B

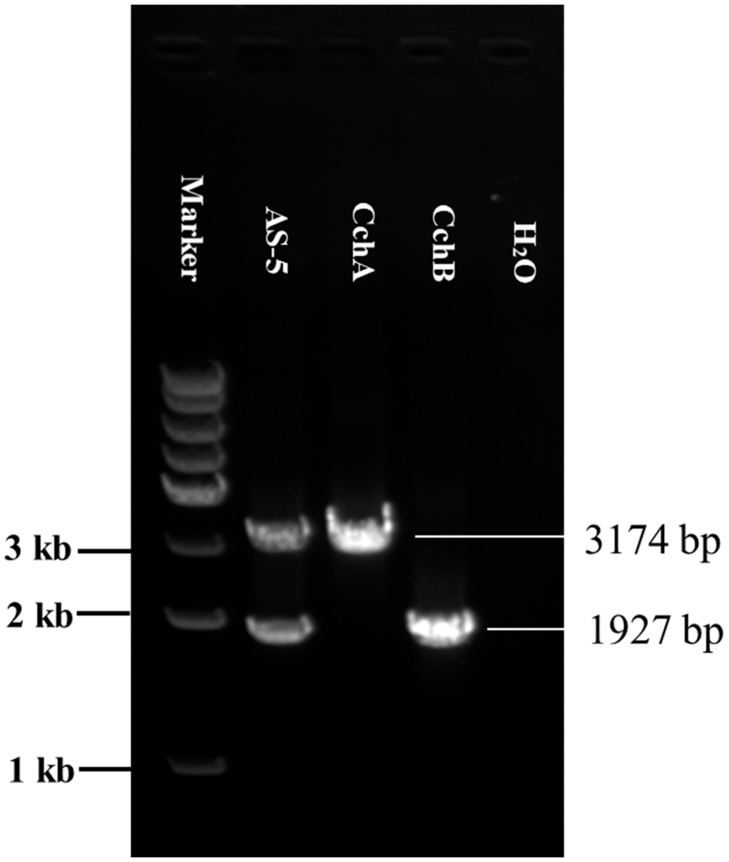

C

Figure S4. PCR verification of *CchA_000467* gene in CchA. (**A**) Primer design sites in CchA. (**B**) Primer design sites in CchB. (**C**) The gel electrophoresis images of the PCR amplification products using the primer pair CchA-F/CchA-R3 in the strains CchA, CchB, and *Agrocybe chaxingu* AS-5.

Figure S5.

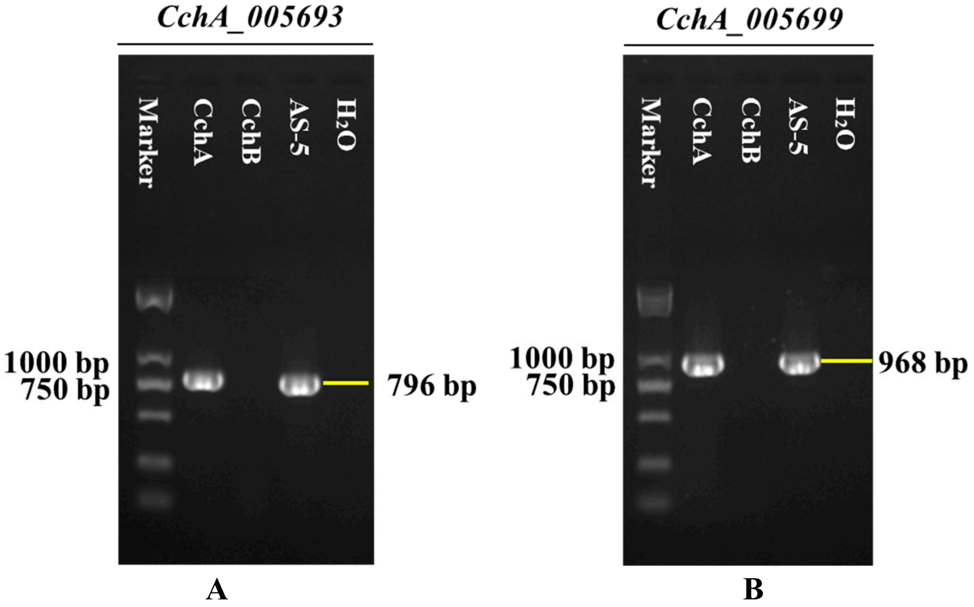

Figure S5. PCR verification of *CchA_005693* and *CchA_005699* unique genes in CchA. (**A**) The gel electrophoresis images of the PCR amplification products using the primer pair 005693F/005693R in the strains CchA, CchB, and *Agrocybe chaxingu* AS-5. (**B**) The gel electrophoresis images of the PCR amplification products using the primer pair 005699F/005693R in the strains CchA, CchB, and *A. chaxingu* AS-5.

Table S1. Information on the 15 species used for constructing the phylogenomic analysis.

**Species**	**Strain**	**Position in classification**	**Source***
Agrocybe chaxingu	CchA	Strophariaceae, Agaricales	This study
Agrocybe chaxingu	CchB	Strophariaceae, Agaricales	This study
Agrocybe chaxingu	MP-N11	Strophariaceae, Agaricales	NCBI: GCA_027627235.1
Cyclocybe aegerita	AAE3	Tubariaceae, Agaricales	[1]
Galerina marginata	CBS 339.88	Hymenogastraceae, Agaricales	[2]
Hypholoma sublateritium	FD-334 SS-4	Strophariaceae, Agaricales	[3]
Psilocybe cubensis	MGC-MH-2018	Hymenogastraceae, Agaricales	[4]
Pholiota conissans	CIRM-BRFM 674	Strophariaceae, Agaricales	NCBI: GCA_015484465.1
Flammula alnicola	AH 47727	Hymenogastraceae, Agaricales	NCBI: GCA_015499995.1
Pholiota molesta	CBS 144467	Strophariaceae, Agaricales	[5]
Psilocybe cf. *subviscida*	CBS 101986	Hymenogastraceae, Agaricales	[6]
Agrocybe pediades	CBS 102.39	Strophariaceae, Agaricales	NCBI: GCA_013053245.1
Psilocybe cyanescens	2631	Hymenogastraceae, Agaricales	[7]
Coprinopsis cinerea	okayama7#130	Psathyrellaceae, Agaricales	[8]
Laccaria bicolor	S238N-H82	Hydnangiaceae, Agaricales	[9]

*: NCBI stands for the National Center for Biotechnology Information.

Table S2. Sequencing data for the genome assembly of CchA and CchB.

Agrocybe chaxingu	Sequencing Strategy	Sequencing Platform	Reads Number	Clean Data (Gb)	Sequence Coverage (X)
CchA	MGI	MGISEQ-T7	31,828,078	9.55	193.26
	PacBio	PacBio Sequel II	430,713	7.30	147.73
	Hi-C	Illumina NovaSeq 6000	34,938,744	5.04	101.89
	RNA	Illumina NovaSeq 6000	49,577,835	7.26	146.98
CchB	MGI	MGISEQ-T7	23,287,534	6.99	138.56
	PacBio	PacBio Sequel II	457,902	7.04	139.55

Table S3. The lengths of the chromosomes in the genomes of CchA and CchB.

Chromosome	CchA<br>Size (bp)	CchB<br>Size (bp)
Chr1	5186933	5175082
Chr2	5092928	5455595
Chr3	4486466	4680988
Chr4	4483789	4370591
Chr5	4194846	3968922
Chr6	3952073	3820519
Chr7	3946830	4269915
Chr8	3803532	3671746
Chr9	3585783	3489017
Chr10	3388903	3544728
Chr11	3146941	3134427
Chr12	2680792	2979525
Chr13	2654686	3098963
Total	50604502	51660018

Table S4. Statistics for non-coding RNA annotation in CchA and CchB.

Type		CchA		CchB	
		**Total length (bp)**	**% of genome**	**Total length (bp)**	**% of genome**
rRNA		32857	0.0649	35313	0.0684
	5.8S	918	0.0018	1071	0.0021
	18S	10422	0.0206	12136	0.0235
	28S	20927	0.0414	21516	0.0416
	5S	590	0.0012	590	0.0011
snRNA		1257	0.0025	1266	0.0024
	splicing	1257	0.0025	1266	0.0024
snoRNA		598	0.0012	598	0.0011
total		34712	0.0686	37177	0.0719

Table S5. Statistics for protein prediction in the genomes of CchA and CchB.

**Method**	**Software**	**Species**	**CchA protein number**	**CchB protein number**
Ab initio	Augustus	—	12640	12639
		—	10176	10493
		—	16328	16542
Homology-based	GeMoMa	*Psilocvbe cubensis*	8561	8747
		Agrocybe pediades	8737	8346
		Pholiota molesta	9093	9101
		Hypholoma sublateritium	8333	8571
RNAseq	PASA	—	23231	21632
tRNA	tRNAscan-SE	—	161	157
Integration	EVM	—	14376	14207

Table S6. CAZyme annotation counts for the CchA and CchB genomes.

CAZy enzymes	CchA genome	CchB genome
auxiliary activities (AAs)	112	108
carbohydrate-binding modules (CBMs)	10	10
carbohydrate esterases (CEs)	45	44
glycoside hydrolases (GHs)	198	194
glycosyl transferases (GTs)	60	59
polysaccharide lyases (PLs)	13	12
Total	438	427

Table S7. Information on the 15 strains of basidiomycetes used for constructing the phylogenetic analysis based on the amino acid sequences of CchA_000467 and its homologs.

**Species**	**Strain**	**Assembly type**	**Source***
Cyclocybe aegerita	AAE3	haploid	[10]
Agrocybe chaxingu	MP-N11	haploid	NCBI: GCA_027627235.1
Hypholoma sublateritium	FD-334 SS-4	haploid	[3]
Pholiota conissans	CIRM-BRFM 674	haploid	NCBI: GCA_015484465.1
Galerina marginata	CBS 339.88	haploid	[2]
Agrocybe chaxingu	CchA	haploid	This study
Agrocybe chaxingu	CchB	haploid	This study
Psilocybe cubensis	MGC-MH-2018	haploid	[4]
Pholiota molesta	CBS 144467	haploid	[5]
Lentinula edodes	sp3	haploid	[11]
Lentinula edodes	sp30	haploid	[11]
Pleurotus pulmonarius	PM_ss13	haploid	NCBI: GCA_012979565.1
leurotus pulmonarius	PM_ss5	haploid	NCBI: GCA_012980535.1
Pleurotus ostreatus	PC9.15	haploid	NCBI: GCA_029852705.2
Pleurotus ostreatus	PC9	haploid	NCBI: GCA_014466165.1

*: NCBI stands for the National Center for Biotechnology Information.

Table S8. The gene clusters comparison of CchA and CchB predicted by antiSMASH.

NO.	Secondary metabolites clusters	CchA		CchB	
		Gene cluster count	Location	Gene cluster count	Location
1	Fungal-RiPP-like	4	Chr1（1）:<br>175714 - 250502 nt. (total: 74789 nt)<br>Chr3（1）:<br>2521492 - 2582846 nt. (total: 61355 nt)<br>Chr8（1）:<br>1047600 - 1108265 nt. (total: 60666 nt)<br>Chr11（1）:<br>224537 - 314349 nt. (total: 89813 nt)	3	Chr1（1）:<br>158482 - 233347 nt. (total: 74866 nt)<br>Chr3（1）:<br>2664558 - 2725912 nt. (total: 61355 nt)<br>Chr11（1）:<br>2795407 - 2858497 nt. (total: 63091 nt)
2	Fungal-RiPP	1	Chr4（1）:<br>2838571 - 2878948 nt. (total: 40378 nt)	1	Chr4（1）:<br>2780641 - 2821003 nt. (total: 40363 nt)
3	Terpene	17	Chr2（4）:<br>852934 - 870067 nt. (total: 17134 nt)<br>1107395 - 1125373 nt. (total: 17979 nt)<br>1272775 - 1289282 nt. (total: 16508 nt)<br>3751870 - 3773203 nt. (total: 21334 nt)<br>Chr5（2）:<br>2679425 - 2695772 nt. (total: 16348 nt)<br>3896639 - 3917913 nt. (total: 21275 nt)<br>Chr6（2）:<br>356102 - 375965 nt. (total: 19864 nt)<br>418166 - 439411 nt. (total: 21246 nt)	20	Chr2（4）:<br>662501 - 678802 nt. (total: 16302 nt)<br>924376 - 943886 nt. (total: 19511 nt)<br>1105184 - 1121727 nt. (total: 16544 nt)<br>3924870 - 3946189 nt. (total: 21320 nt)<br>Chr6（2）:<br>201966 - 220196 nt. (total: 18231 nt)<br>262889 - 284154 nt. (total: 21266 nt)<br>Chr7（1）:<br>1730222 - 1748423 nt. (total: 18202 nt)

			Chr7（1）:<br>1896495 - 1914694 nt. (total: 18200 nt)		Chr5（2）:<br>2206551 - 2222898 nt. (total: 16348 nt)<br>3609272 - 3630550 nt. (total: 21279 nt)
			Chr8（4）:<br>137491 - 155983 nt. (total: 18493 nt)<br>296127 - 311653 nt. (total: 15527 nt)<br>1740201 - 1755723 nt. (total: 15523 nt)<br>3508979 - 3531483 nt. (total: 22505 nt)		Chr8（5）:<br>28993 - 47287 nt. (total: 18295 nt)<br>135595 - 136923 nt. (total: 1329 nt)<br>201967 - 218141 nt. (total: 16175 nt)<br>1468932 - 1487297 nt. (total: 18366 nt)<br>3471055 - 3492336 nt. (total: 21282 nt)
			Chr10（2）:<br>673080 - 689781 nt. (total: 16702 nt)<br>1182103 - 1203451 nt. (total: 21349 nt)		Chr10（3）:<br>98195 - 113100 nt. (total: 14906 nt)<br>765087 - 781391 nt. (total: 16305 nt)<br>1210008 - 1231356 nt. (total: 21349 nt)
			Chr11（1）:<br>1158350 - 1175473 nt. (total: 17124 nt)		Chr9（1）:<br>2917966 - 2939311 nt. (total: 21346 nt)
			Chr12（1）:<br>2268377 - 2290038 nt. (total: 21662 nt)		Chr11（1）:<br>1897624 - 1918912 nt. (total: 21289 nt)
					Chr12（1）:<br>2483176 - 2504836 nt. (total: 21661 nt)
4	NRPS	1	Chr2（1）:<br>1748876 - 1796845 nt. (total: 47970 nt)	1	Chr2（1）:<br>1606041 - 1654010 nt. (total: 47970 nt)
5	NRPS-like	4	Chr3（1）:<br>4174823 - 4230310 nt. (total: 55488 nt)<br>Chr7（1）:<br>1462680 - 1506607 nt. (total: 43928 nt)<br>Chr9（1）:<br>1518780 - 1572585 nt. (total: 53806 nt)	4	Chr3（1）:<br>4253913 - 4308264 nt. (total: 54352 nt)<br>Chr7（1）:<br>1368797 - 1412722 nt. (total: 43926 nt)<br>Chr9（1）:<br>2126032 - 2178922 nt. (total: 52891 nt)

			Chr13（1）:<br>1677303 - 1721988 nt. (total: 44686 nt)		Chr13（1）:<br>1021380 - 1066065 nt. (total: 44686 nt)
6	NI-siderophore	1	Chr5（1）:<br>1941032 - 1956644 nt. (total: 15613 nt)	1	Chr5（1）：<br>1721756 - 1737384 nt. (total: 15629 nt)
7	Indole	2	Chr5（1）:<br>2731717 - 2753128 nt. (total: 21412 nt)<br>Chr13（1）:<br>1972692 - 1994123 nt. (total: 21432 nt)	1	Chr13（1）:<br>748140 - 769562 nt. (total: 21423 nt)
8	T1PKS	1	Chr9（1）:<br>3,190,389 - 3,239,556 nt. (total: 49,168 nt)	1	Chr9（1）:<br>269680 - 318853 nt. (total: 49174 nt)
9	Terpene, T1PKS	1	Chr7（1）:<br>3345733 - 3411927 nt. (total: 66195 nt)	1	Chr7（1）:<br>3208034 - 3274232 nt. (total: 66199 nt)
10	T1PKS, Terpene, 2NRPS-like	1	Chr11（1）:<br>1297823 - 1359498 nt. (total: 61676 nt)		
11	T1PKS, 2NRPS-like, Terpene			1	Chr11（1）:<br>1630006 - 1691672 nt. (total: 61667 nt)
Total		33		34	

Table S9. Significant GO enrichment results for the rearranged genes of CchA.

Term Name	MainClass	GeneHitsInSelectedSet	AllGenesInBackground	corrected p-value(BH method)
meiotic spindle pole body	Cellular Component	3	3298	0.00321607
LINC complex	Cellular Component	2	3298	0.00321607
integral component of nuclear inner membrane	Cellular Component	2	3298	0.00321607
ribonucleoside-diphosphate reductase complex	Cellular Component	2	3298	0.00321607
intrinsic component of nuclear inner membrane	Cellular Component	2	3298	0.00321607
microtubule organizing center attachment site	Cellular Component	2	3298	0.00321607
transcriptional activator activity, RNA polymerase II transcription factor binding	Molecular Function	2	2954	0.004296943
ribonucleoside-diphosphate reductase activity, thioredoxin disulfide as acceptor	Molecular Function	2	2954	0.004296943
oxidoreductase activity, acting on CH or CH2 groups, disulfide as acceptor	Molecular Function	2	2954	0.004296943
CDP reductase activity	Molecular Function	2	2954	0.004296943
ribonucleoside-diphosphate reductase activity	Molecular Function	2	2954	0.004296943
transcriptional repressor activity, RNA polymerase II activating transcription factor binding	Molecular Function	2	2954	0.004296943
nuclear membrane part	Cellular Component	2	3298	0.005490967
meiotic spindle	Cellular Component	3	3298	0.006947719
oxidoreductase activity, acting on CH or CH2 groups	Molecular Function	2	2954	0.007332928
inner plaque of spindle pole body	Cellular Component	2	3298	0.00794305

interphase microtubule organizing center	Cellular Component	2	3298	0.00794305
inner plaque of mitotic spindle pole body	Cellular Component	2	3298	0.00794305
new mitotic spindle pole body	Cellular Component	2	3298	0.00794305
establishment of spindle pole body localization	Biological Process	2	3403	0.011479365
establishment of spindle pole body localization to nuclear envelope	Biological Process	2	3403	0.011479365
dCDP biosynthetic process	Biological Process	2	3403	0.011479365
pyrimidine ribonucleoside diphosphate metabolic process	Biological Process	2	3403	0.011479365
dCDP metabolic process	Biological Process	2	3403	0.011479365
CDP metabolic process	Biological Process	2	3403	0.011479365
spindle pole body localization	Biological Process	2	3403	0.011479365
spindle pole body localization to nuclear envelope	Biological Process	2	3403	0.011479365
centromere localization	Biological Process	2	3403	0.011479365
centromere clustering at the nuclear envelope	Biological Process	2	3403	0.011479365
centromere clustering	Biological Process	2	3403	0.011479365
pyrimidine nucleoside diphosphate metabolic process	Biological Process	2	3403	0.013237197
pyrimidine nucleoside diphosphate biosynthetic process	Biological Process	2	3403	0.013237197
deoxyribonucleoside diphosphate metabolic process	Biological Process	2	3403	0.013237197
deoxyribonucleoside diphosphate biosynthetic process	Biological Process	2	3403	0.013237197
pyrimidine deoxyribonucleoside diphosphate	Biological Process	2	3403	0.013237197

metabolic process				
pyrimidine deoxyribonucleoside diphosphate biosynthetic process	Biological Process	2	3403	0.013237197
deoxyribonucleoside triphosphate biosynthetic process	Biological Process	2	3403	0.013237197
pyrimidine deoxyribonucleoside triphosphate biosynthetic process	Biological Process	2	3403	0.013237197
spindle pole body	Cellular Component	4	3298	0.013267221
old mitotic spindle pole body	Cellular Component	2	3298	0.015527085
spindle pole	Cellular Component	4	3298	0.015527085
microtubule organizing center	Cellular Component	4	3298	0.016755767
nucleotide binding	Molecular Function	8	2954	0.017463362
nucleoside phosphate binding	Molecular Function	8	2954	0.017463362
transcriptional repressor activity, RNA polymerase II transcription factor binding	Molecular Function	2	2954	0.017723562
protein anchor	Molecular Function	2	2954	0.01960404
NADP binding	Molecular Function	2	2954	0.01960404
deoxyribonucleoside triphosphate metabolic process	Biological Process	2	3403	0.019878734
pyrimidine deoxyribonucleoside triphosphate metabolic process	Biological Process	2	3403	0.019878734
nuclear inner membrane	Cellular Component	2	3298	0.0201467
nucleoside diphosphate biosynthetic process	Biological Process	2	3403	0.0222716
pyrimidine nucleoside triphosphate biosynthetic process	Biological Process	2	3403	0.0222716
pyrimidine deoxyribonucleotide metabolic	Biological Process	2	3403	0.0222716

process				
pyrimidine deoxyribonucleotide biosynthetic process	Biological Process	2	3403	0.0222716
deoxyribonucleotide biosynthetic process	Biological Process	2	3403	0.0222716
2'-deoxyribonucleotide biosynthetic process	Biological Process	2	3403	0.0222716
deoxyribose phosphate biosynthetic process	Biological Process	2	3403	0.0222716
ATP binding	Molecular Function	6	2954	0.024580712
adenyl ribonucleotide binding	Molecular Function	6	2954	0.024580712
adenyl nucleotide binding	Molecular Function	6	2954	0.024580712
small molecule binding	Molecular Function	8	2954	0.024580712
ribonucleotide binding	Molecular Function	7	2954	0.026364878
microtubule organizing center part	Cellular Component	2	3298	0.026767076
carbohydrate derivative binding	Molecular Function	7	2954	0.028327693
RNA helicase activity	Molecular Function	3	2954	0.028327693
ATP-dependent RNA helicase activity	Molecular Function	3	2954	0.028327693
RNA-dependent ATPase activity	Molecular Function	3	2954	0.028327693
pyrimidine nucleoside triphosphate metabolic process	Biological Process	2	3403	0.029981281
mitotic spindle pole body	Cellular Component	3	3298	0.03298419
drug binding	Molecular Function	6	2954	0.034299914
anion binding	Molecular Function	8	2954	0.034299914
spindle	Cellular Component	4	3298	0.03465158
deoxyribonucleotide metabolic process	Biological Process	2	3403	0.036078477
2'-deoxyribonucleotide metabolic process	Biological Process	2	3403	0.036078477
deoxyribose phosphate metabolic process	Biological Process	2	3403	0.036078477
flavin adenine dinucleotide binding	Molecular Function	2	2954	0.043355257

epoxide hydrolase activity	Molecular Function	1	2954	0.043355257
ether hydrolase activity	Molecular Function	1	2954	0.043355257
protein self-association	Molecular Function	1	2954	0.043355257
protein kinase activity involved in regulation of protein localization to cell division site involved in cytokinesis	Molecular Function	1	2954	0.043355257

Table S10. Significant KEGG enrichment results for the unique genes in CchA.

Term Name	MainClass	GeneHitsInSelectedSet	AllGenesInBackground	enrichFactor	corrected p-value(BH method)
DNA replication	Genetic Information Processing	3	4183	32.42635659	0.001466041
RNA degradation	Genetic Information Processing	2	4183	15.23861566	0.025384396

Table S11. Summary of the RNA-seq data utilized in this study.

**Sample***	**No. of raw reads**	**Total bases of raw reads**	**No. of clean reads**	**Total bases of clean reads**
MY1	45,203,170	6,780,475,500	44,565,584	6,592,150,152
MY2	43,549,648	6,532,447,200	42,947,792	6,406,641,914
MY3	45,697,900	6,854,685,000	45,069,624	6,726,140,297
FB1	43,812,964	6,621,587,500	43,159,697	6,445,285,621
FB2	40,102,182	6,245,169,200	39,632,417	6,102,114,782
FB3	41,463,126	6,382,514,300	40,896,612	6,235,524,149

*: MY: Vegetative mycelia of the strain *Agrocybe chaxingu* AS-5 on PDA medium; FB: Mature fruiting bodies of the *A. chaxingu* AS-5 strain.

Table S12. Unique genes expressed only in the fruiting body stage in CchA and CchB.

**Strain**	**CchA**	**CchB**
Gene	*CchA_007304*	*CchB_006750*
	CchA_007108	*CchB_008453*
	CchA_004881	*CchB_013785*
	CchA_004042	*CchB_012829*
	CchA_003315	*CchB_008476*
	CchA_011148	*CchB_007110*
	CchA_001317	*CchB_009098*
	CchA_003137	*CchB_011358*
	CchA_004527	*CchB_003173*
	CchA_010037	*CchB_013389*
	CchA_011182	*CchB_003333*
	CchA_002997	*CchB_011949*
	CchA_013297	*CchB_011365*
	CchA_007221	*CchB_000013*
	CchA_009782	*CchB_013937*
	CchA_009790	
	CchA_008308	
	CchA_014331	
	CchA_008110	
	CchA_001598	
	CchA_012899	
	CchA_007364	
	CchA_012653	
	CchA_003490	

References

1. Gupta, D.K.; Rühl, M.; Mishra, B.; Kleofas, V.; Hofrichter, M.; Herzog, R.; Pecyna, M.J.; Sharma, R.; Kellner, H.; Hennicke, F.; et al. The genome sequence of the commercially cultivated mushroom *Agrocybe aegerita* reveals a conserved repertoire of fruiting-related genes and a versatile suite of biopolymer-degrading enzymes. *BMC Genom.* **2018**, *19*, 48.
2. Riley, R.; Salamov, A.A.; Brown, D.W.; Nagy, L.G.; Floudas, D.; Held, B.W.; Levasseur, A.; Lombard, V.; Morin, E.; Otillar, R.; et al. Extensive sampling of basidiomycete genomes demonstrates inadequacy of the white-rot/brown-rot paradigm for wood decay fungi. *Proc. Natl. Acad. Sci. USA* **2014**, *111*, 9923–9928.
3. Kohler, A.; Kuo, A.; Nagy, L.G.; Morin, E.; Barry, K.W.; Buscot, F.; Canbäck, B.; Choi, C.; Cichocki, N.; Clum, A.; et al. Convergent losses of decay mechanisms and rapid turnover of symbiosis genes in mycorrhizal mutualists. *Nature Genet.* **2015**, *47*, 410–415.
4. McKernan, K.; Kane, L.T.; Crawford, S.; Chin, C.S.; Trippe, A.; McLaughlin, S. A draft reference assembly of the *Psilocybe cubensis* genome. *F1000Research* **2021**, 10, 281.

5. Steindorff, A.S.; Carver, A.; Calhoun, S.; Stillman, K.; Liu, H.; Lipzen, A.; He, G.; Yan, M.; Pangilinan, J.; LaButti, K.; et al. Comparative genomics of pyrophilous fungi reveals a link between fire events and developmental genes. *Environ. Microbiol.* **2021**, 23, 99–109.
6. Floudas, D.; Bentzer, J.; Ahrén, D.; Johansson, T.; Persson, P.; Tunlid, A. Uncovering the hidden diversity of litter-decomposition mechanisms in mushroom-forming fungi. *ISME J.* **2020**, 14, 2046–2059.
7. Yan, L.Y.; Wang, Z.H.; Song, W.D.; Fan, P.M.; Kang, Y.P.; Lei, Y.; Wan, L.Y.; Huai, D.X.; Chen, Y.N.; Wang, X.; et al. Genome sequencing and comparative genomic analysis of highly and weakly aggressive strains of *Sclerotium rolfsii*, the causal agent of peanut stem rot. *BMC Genomics* **2021**, 22, 276.
8. Stajich, J.E.; Wilke, S.K.; Ahrén, D.; Au, C.H.; Birren, B.W.; Borodovsky, M.; Burns, C.; Canbäck, B.; Casselton, L.A.; Cheng, C.K.; et al. Insights into evolution of multicellular fungi from the assembled chromosomes of the mushroom *Coprinopsis cinerea* (*Coprinus cinereus*). *Proc. Natl. Acad. Sci. USA* **2010**, *107*, 11889–11894.
9. Martin, F.; Aerts, A.; Ahrén, D.; Brun, A.; Danchin, E.G.J.; Duchaussoy, F.; Gibon, J.; Kohler, A.; Lindquist, E.; Pereda, V.; et al. The genome of *Laccaria bicolor* provides insights into mycorrhizal symbiosis. *Nature* **2008**, *452*, 88–92.
10. Walker, B.J.; Abeel, T.; Shea, T.; Priest, M.; Abouelliel, A.; Sakthikumar, S.; Cuomo, C.A.; Zeng, Q.D.; Wortman, J.; Young, S.K.; et al. Pilon: An integrated tool for comprehensive microbial variant detection and genome assembly improvement. *PLoS ONE* **2014**, *9*, e112963.
11. Pardo, E.H.; O'Shea, S.F.; Casselton, L.A. Multiple versions of the A mating type locus of *Coprinus cinereus* are generated by three paralogous pairs of multiallelic homeobox genes. *Genetics* **1996**, *144*, 87–94.
